# Challenging the Efficacy of Routine Antibiotic Skin Testing: Insights From Two Cases of Anaphylaxis

**DOI:** 10.7759/cureus.84729

**Published:** 2025-05-24

**Authors:** Jefferson Daniel, Rashmitha Thippaiah, Devasahayam J Christopher

**Affiliations:** 1 Department of Pulmonary Medicine, Christian Medical College Vellore, Vellore, IND

**Keywords:** allergy and anaphylaxis, allergy label, allergy skin testing, antibiotic allergy, drug allergy, intradermal tests, penicillin allergy, preemptive allergy testing

## Abstract

This series presents two cases of anaphylaxis following the administration of beta-lactam antibiotics, piperacillin-tazobactam, and cefotaxime in patients who had previously shown negative skin test results. A 32-year-old woman with no history of allergies developed anaphylaxis 15 minutes after receiving piperacillin-tazobactam, despite a negative intradermal test. Similarly, a 48-year-old woman with no prior allergic response experienced anaphylaxis within 10 minutes of cefotaxime administration even after a negative intradermal test. Both patients were successfully treated with epinephrine, fluids, and corticosteroids with close monitoring of further complications. These cases highlight the limitations of preemptive skin testing for antibiotics, a practice still commonly followed in certain healthcare settings in India, despite growing concerns about its predictive reliability and lack of standardization. Unstandardized methods of skin testing, as commonly practiced in India, have poor sensitivity and are unreliable for predicting drug-related anaphylaxis. Furthermore, even small intravenous test doses can trigger severe anaphylactic reactions, underscoring the need for caution during antibiotic allergy testing. According to the European Academy of Allergy and Clinical Immunology (EAACI) and the American Academy of Allergy, Asthma, and Immunology (AAAAI) guidelines, skin testing is recommended only in suspected cases of antibiotic allergy, with diagnostic algorithms tailored to the nature of the reaction. However, these guidelines are not universally followed, particularly in regions where standardized testing resources and training are limited. These cases emphasize the need for standardized indications and protocols for skin testing in India, along with the adaptation of international guidelines to suit India’s unique clinical and healthcare context, potentially serving as a model for other resource-limited countries that face similar challenges.

## Introduction

Anaphylaxis is a severe, potentially life-threatening, IgE-mediated hypersensitivity reaction that can occur rapidly after exposure to an allergen. In medical settings, drug-induced anaphylaxis is a significant concern, particularly with beta-lactam antibiotics, which are among the most frequently implicated classes of drugs [1]. This series describes two cases of anaphylaxis following piperacillin-tazobactam and cefotaxime administrations in patients with negative skin test results. International guidelines, such as those from the European Academy of Allergy and Clinical Immunology (EAACI), advise that antibiotic skin testing should be reserved for patients with a well-documented history suggestive of allergy. Global recommendations highlight the use of standardized procedures, including validated non-irritant drug concentrations, and rely on the availability of neat drug preparations free of additives, trained allergy professionals, and tailored diagnostic pathways [2].

In many high-income countries, the assessment of antibiotic allergies, particularly penicillin allergies, is guided by a structured, specialist-led approach. It typically involves a thorough medical history, review of clinical documentation, standardized skin testing, and confirmatory drug challenges, all supported by established clinical protocols and national guidelines [3]. In contrast, practices in India and other low-resource settings often lack such standardization. Skin testing procedures vary significantly, with inconsistent drug concentrations, limited availability of validated reagents, inadequate documentation, and use of preemptive testing without clear indications [3]. A recent review of penicillin allergy management in India and Sri Lanka further underscored the absence of national guidelines and highlighted systemic challenges, including insufficient training and an underdeveloped infrastructure [4]. Similarly, in China, inappropriate testing practices have contributed to the mislabeling of patients as allergic, leading to unnecessary avoidance of beta-lactam antibiotics and complicating antimicrobial stewardship efforts [5]. These disparities reflect a pressing need for investment in local capacity, the development of context-specific protocols, and improved access to diagnostic resources to enhance the safe and effective management of antibiotic allergies in these regions.

## Case presentation

Case study 1

A 32-year-old woman with a history of nephrolithiasis and recurrent culture-confirmed urinary tract infections (UTIs), previously treated with oral antibiotics that she tolerated well, was scheduled for nephrolithotomy under general anesthesia. The patient had no history of drug allergies or anaphylactic responses. Piperacillin-tazobactam was administered as part of the hospital’s standard preoperative protocol for infection prophylaxis, consistent with the local antimicrobial stewardship guidelines and the patient’s history of recurrent UTIs. At the referring hospital, both a skin prick test and an intradermal test were performed using the injectable formulation of piperacillin-tazobactam in accordance with their preoperative protocol. The patient exhibited no reaction to either test at the 30-minute mark. She was not on any systemic or topical medications, such as antihistamines, tricyclic antidepressants, or corticosteroids, which could have interfered with the interpretation of the results. However, the concentrations used for skin testing have not been documented. Following the negative skin test results, piperacillin-tazobactam 4.5 g was administered as an intravenous infusion, as per protocol. Approximately 15 minutes after the infusion was initiated, and while it was still ongoing, the patient developed signs of anaphylaxis, including tachycardia, shortness of breath, throat tightness, urticaria, and hypotension (systolic blood pressure of 70 mmHg). Immediate treatment was initiated with a single dose of intramuscular epinephrine (0.5 mg), intravenous fluids, and corticosteroids, resulting in rapid symptomatic improvement. Owing to persistent hypotension, a brief noradrenaline infusion was administered for a few hours. No surgery was performed. A few months later, the patient underwent a planned allergy evaluation at our center. Based on the drug cross-reactivity charts and hospital antimicrobial stewardship policy, meropenem was identified as a suitable alternative to piperacillin-tazobactam. At the request of both the patient and the anesthesiologist, a supervised graded challenge with meropenem was performed to confirm tolerance prior to proceeding with surgery. The surgery was subsequently performed successfully, and the patient was discharged uneventfully with instructions to avoid beta-lactam antibiotics that are known to have documented cross-reactivity.

Case study 2

A 48-year-old woman with no known drug allergies was scheduled for elective hysterectomy for symptomatic uterine fibroids. As per the protocol at the hospital where she was initially evaluated, both a skin prick test and an intradermal test were performed using cefotaxime, yielding negative results at 30 minutes. However, the concentrations used for testing were not documented. Following negative skin test results, a 1-g intravenous bolus dose of cefotaxime was administered as per the standard protocol. Within 10 minutes of administration, the patient developed signs and symptoms of anaphylaxis, including tachycardia, hypotension, pruritus, respiratory distress, and widespread urticaria. Immediate treatment was initiated with two intramuscular doses of epinephrine (0.5 mg each), along with intravenous fluids and corticosteroids, leading to the resolution of symptoms. Surgery was subsequently postponed. A few months later, she visited our emergency department with an acute febrile illness and was diagnosed with severe community-acquired pneumonia. The allergy service was consulted before administering a suitable antibiotic owing to the fear of recurrent life-threatening anaphylaxis. Based on the known cross-reactivity patterns described in the literature, we recommended meropenem. A supervised graded challenge with meropenem was performed in the ICU, and the patient tolerated the full dose without any adverse reactions. The patient eventually recovered and was discharged with instructions to avoid beta-lactam antibiotics with a documented cross-reactivity.

Both cases were classified as Grade III anaphylaxis according to the World Allergy Organization criteria, given the presence of hypotension, respiratory symptoms, and the need for vasopressor support. Each patient required subsequent ICU monitoring for 24 hours. No biphasic reactions were observed during the observation period. Both patients remained free of allergic reactions during the two years of consistent follow-up.

## Discussion

**Figure 1:**
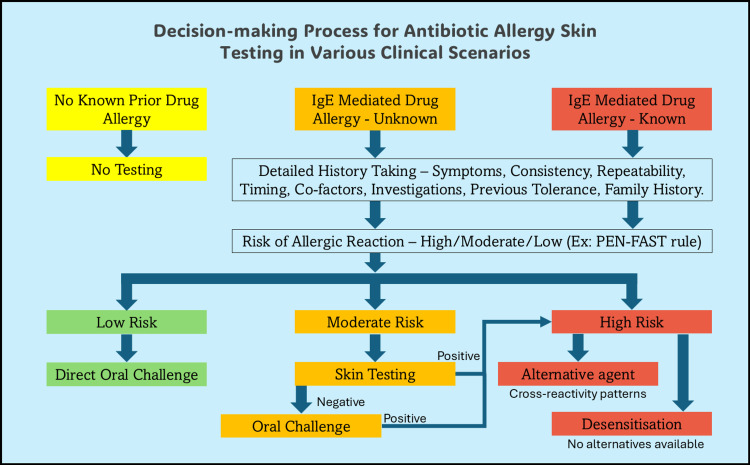
Decision-making algorithm for antibiotic allergy skin testing in diverse clinical contexts: a resource-conscious approach In resource-limited settings, skin testing in high-risk cases is generally avoided due to a lack of adequate infrastructure. Testing may be considered on a case-by-case basis, guided by clinical judgment and patient safety. Note: Clinical training is essential before undertaking any drug challenge procedures. Image Credits: Authors' original creation.

Antibiotic allergy skin testing plays a well-established role in guiding antibiotic administration, particularly in ruling out IgE-mediated penicillin allergies, by detecting drug-specific IgE antibodies that mediate immediate hypersensitivity reactions. Research shows that only 72.2% of patients with a documented history of penicillin allergy, including IgE-mediated reactions such as anaphylaxis, urticaria, or angioedema, test positive on skin testing [6]. This indicates that while skin testing is valuable for confirming true drug allergies, it has limited sensitivity (penicillins, 30%; cephalosporins, 0%), although it is highly specific (95%) and cannot predict all allergic reactions [7,8]. Skin prick testing for beta-lactam allergy may yield false-negative results because of the degradation of specific IgE over time and the involvement of non-IgE-mediated mechanisms, such as direct mast cell activation, which are not detectable by conventional testing [9,10]. Additionally, the lack of standardized testing concentrations for many beta-lactams limits diagnostic accuracy. Nevertheless, it remains a useful tool in the management of patients with documented antibiotic allergies, aiding specialists in de-labeling or selecting suitable alternative antibiotics [11]. In cases where routine skin testing is inconclusive or infeasible, alternative risk-stratification methods, such as detailed clinical history, validated risk assessment tools, and supervised drug provocation testing, should be prioritized (Figure 1). Given these limitations, there is a need to revise the current clinical practices by promoting physician education on drug allergy evaluation, updating institutional allergy testing protocols, and integrating multidisciplinary approaches to optimize drug allergy management and antibiotic stewardship. 

In countries such as India, China, and Sri Lanka, routine preemptive skin testing is commonly performed before the administration of the first dose of an antibiotic [4,12,13]. Routine penicillin skin testing is associated with a high false-positive rate, reported to be as high as 97% in some studies, which may contribute to increased antimicrobial resistance by encouraging the unnecessary use of broad-spectrum antibiotics [12,14]. The absence of standardized protocols for antibiotic skin testing increases the risk of misdiagnosis, delayed treatment, and inappropriate selection of antibiotics. Our cases underscore the potential for severe allergic reactions to occur even after skin tests for antibiotics are negative. 

Routine skin antibiotic testing poses several challenges. In emergency situations such as sepsis, in which early antibiotic administration is critical, these practices can cause significant delays. A routine skin test protocol that involves skin prick testing, followed by intradermal testing, typically includes a 15-20-minute observation period after each step for immediate reactions, followed by a limited time of monitoring to detect any delayed systemic responses, particularly in high-risk patients [15]. This conflicts with the recommended "golden hour," the first hour after sepsis recognition, during which prompt antibiotic administration is crucial for improving survival outcomes [16]. While routine skin testing is not universally practiced in emergency settings, in some institutions, particularly in parts of Asia, it may still be performed because of medicolegal concerns [4,17]. Second, antibiotic skin testing demonstrates poor sensitivity for detecting immediate hypersensitivity reactions, with only 28% of patients with documented drug allergies testing positive in one study [9]. This finding suggests that a negative skin test result does not reliably exclude the risk of anaphylaxis. Additionally, skin test positivity declines over time, reducing its reliability when the initial allergic reaction occurred more than 10 years prior [9]. Test doses are also ineffective in screening for delayed hypersensitivity reactions, such as Stevens-Johnson syndrome or drug reactions with eosinophilia and systemic symptoms (DRESS), which can be life-threatening [9]. The lack of commercially available skin test kits for penicillin allergies in India further complicates this process [4]. Finally, even a small fraction of an intravenous drug administered as a test dose can potentially trigger fatal anaphylaxis in IgE-mediated reactions, making the use of this approach in general practice inherently risky [5]. The EAACI and the American Academy of Allergy, Asthma, and Immunology (AAAAI) provide recommendations for skin testing only in suspected cases of antibiotic allergies. Routine antibiotic skin testing is not a standard practice in most European countries, except for high-risk cases, in line with the EAACI recommendations. For a suspected history of beta-lactam hypersensitivity, the EAACI guidelines suggest a risk stratification approach based on index reaction characteristics, including timing and severity [18]. The guidelines recommend distinct diagnostic algorithms for high-risk and low-risk patients, with immediate reactions occurring within 1-6 hours and non-immediate reactions occurring more than one hour after the initial drug administration. Interestingly, updated guidelines indicate that for some low-risk, non-immediate reactions, especially in children, skin tests may not be mandatory [18]. This represents a shift from previous practices and highlights the evolving nature of diagnostic approaches to antibiotic allergies. These guidelines recommend specific skin test concentrations to achieve a specificity of at least 95% [2]. However, further research is needed to recommend appropriate concentrations of many drugs, emphasizing the need for further multicenter studies to establish and validate standardized protocols for drug allergy-related skin testing.

## Conclusions

This case series highlights the urgent need to reconsider the routine practice of preemptive skin testing for antibiotics, particularly beta-lactams. Despite the negative skin test results, both patients experienced severe anaphylactic reactions, underscoring the limited sensitivity and predictive value of unstandardized tests. These cases also underscore the importance of maintaining high clinical vigilance during antibiotic administration, particularly in patients with prior drug exposure or other risk factors for hypersensitivity. In addition, these findings support the implementation of standardized antibiotic administration protocols that emphasize patient-specific risk assessment over routine preemptive testing. There is also a critical need to revise and adapt international guidelines to the Indian healthcare context to ensure alignment with local epidemiology, healthcare infrastructure, and clinical practices. To drive meaningful changes, national policy frameworks should be updated to reflect these priorities. However, it is important to acknowledge that these conclusions were based on a limited number of cases. Larger, multicenter studies are essential to validate these observations and inform evidence-based policy and clinical guidelines. Future research should focus on establishing validated drug concentrations for skin testing, developing standardized diagnostic algorithms, incorporating the basophil activation test, and exploring alternative diagnostic approaches, such as in vitro assays for antibiotic allergy assessment in the Indian population.
